# The COVID-19 Vaccination Behavior and Correlates in Diabetic Patients: A Health Belief Model Theory-Based Cross-Sectional Study in China, 2021

**DOI:** 10.3390/vaccines10050659

**Published:** 2022-04-22

**Authors:** Lingrui Duan, Ying Wang, Haoyu Dong, Congying Song, Jinping Zheng, Jing Li, Mufan Li, Jiayu Wang, Jianzhou Yang, Junjie Xu

**Affiliations:** 1 School of Epidemiology and Public Health, Shanxi Medical University, Taiyuan 032000, China; dlr0875@b.sxmu.edu.cn (L.D.); wangying@b.sxmu.edu.cn (Y.W.); lmf98@b.sxmu.edu.cn (M.L.); jiayu12@b.sxmu.edu.cn (J.W.); 2 Department of Endocrinology, Heping Hospital, Changzhi Medical College, Changzhi 046000, China; czmcdhy@czmc.com; 3 Clinical Research Academy, Peking University Shenzhen Hospital, Peking University, Shenzhen 518000, China; songcongying@pkuszh.com; 4 Department of Public Health and Preventive Medicine, Changzhi Medical College, Changzhi 046000, China; zhengjp@czmc.edu.cn; 5 Renal Division, Peking University Shenzhen Hospital, Peking University, Shenzhen 518000, China; lijingp0677@pkuszh.com

**Keywords:** diabetes, COVID-19 vaccine, health belief model, vaccination behavior

## Abstract

The population with diabetes is more susceptible to severe acute respiratory syndrome-associated coronavirus (SARS-CoV)-2, and have a significantly higher coronavirus disease-2019 (COVID-19) mortality rate. Previous studies have shown low willingness for the COVID-19 vaccination, and there are limited reports on the behavior and relevance of the COVID-19 vaccination. This study aimed to determine the uptake behavior and associated factors of the COVID-19 vaccine. In our cross-sectional questionnaire-based clinical study, 645 diabetes patients affiliated with two affiliated hospitals of Changzhi Medical College completed the questionnaire between June to October 2021. The health belief model (HBM) was used in examining factors influencing vaccination behavior. After adjusting for covariates with significant differences in social background characteristics, a multivariable logistic regression was used to determine predictors related to uptake in COVID-19 vaccination. A total of 162 vaccinated and 483 unvaccinated eligible diabetic patients were recruited. Patients who believed that the COVID-19 syndrome is severe (aOR3.67, 95%CI 1.88–7.17; *p* < 0.001), believe that vaccination can significantly reduce the risk of SARS-Cov-2 infection (aOR3.48, 95%CI 1.80–6.73; *p* < 0.001), believe that vaccination is beneficial to themselves and others (aOR 4.53, 95%CI 1.71–11.99; *p* = 0.002), think that relatives’ vaccination status has a positive impact on their vaccination behavior (aOR 5.68, 95%CI 2.83–11.39; *p* < 0.001), and were more likely to be vaccinated; worrying about the adverse health effects of COVID-19 vaccination (aOR 0.18, 95%CI 0.09–0.35; *p* < 0.001) was negatively correlated with COVID-19 vaccination behavior. Health care workers should provide targeted informative interventions based on the safety and protective effects theory of HBM to improve vaccination behavior in patients with diabetes.

## 1. Introduction

The COVID-19 epidemic has brought major challenges to global health. Individuals with underlying diseases such as diabetes are more likely to become critically ill patients with COVID-19 [1,2,3]. Studies have shown that diabetes was associated with disease severity and mortality in COVID-19 patients [4,5]. This may be due to systemic inflammatory responses and impaired immune system function [6] leading to elevated levels of proinflammatory cytokines [7], as well as a pre-existing metabolic inflammatory state in patients with type 2 diabetes mellitus (T2DM) that complicates and prolongs lung injury [8]. A study on the efficacy of the COVID-19 vaccine in the elderly, including the diabetics, showed that the hospitalization rate and case fatality rate of the vaccine group decreased by 55% compared with the non-vaccine group two weeks after being vaccinated against COVID-19 [9]. The World Health Organization (WHO) also recommends patients with chronic diseases, including those with diabetes, receive COVID-19 vaccination against the infection of SARS-CoV-2 [10]. In this global COVID-19 outbreak, diabetes has become one of the most prominent and prevalent risk factors for severe disease course and high mortality in this pandemic [11]. If these diabetic patients are not vaccinated against the COVID-19 in time, their lives and health would be greatly threatened.

The rapidly evolving COVID-19 has increased the difficulty of epidemic prevention and control. Through COVID-19 vaccination and other comprehensive measures, the spread of COVID-19 [12] and the occurrence of severe disease after SARS-CoV-2 infection can be effectively decreased [13]. Vaccination usually has certain benefits in reducing the risk of severe SARS-CoV-2 infection and fatality according to published literature [14], but there were still concerns about the potential harmfulness of COVID-19 vaccination in some patients with diabetes in China (56.4%) [15], Saudi Arabia (29.0%) [16], Malaysia (24.7%) [17] and Italy (14.2%) [18]. The main factors correlated to the willingness to vaccinate against COVID-19 include fear of the adverse reactions to COVID-19 vaccination [17], uncertainty about the genetic composition of COVID-19 vaccines [16], and concern about the safety of COVID-19 vaccines [18]. Previous studies had demonstrated a significant positive correlation between COVID-19 vaccination willingness and vaccination uptake behavior in patients with T2DM [19]. Groups of chronically ill patients, including those with diabetes, have lower COVID-19 vaccination rates, such as 8.3% in the U.S. and 18% in France [20,21]. However, the general population of patients with chronic diseases varies greatly by disease type, resulting in a limited representation of the behaviors of the massive number of diabetic patients. The scale of diabetic patients is as large as 537 million globally, and 141 million in China [22], which raised the concern of studying the COVID-19 vaccination uptake behavior and influencing factors of the diabetics who have already received the COVID-19 vaccine.

While limited behavioral surveys on the vaccination of patients with diabetes were reported [16,18], the factors associated with the vaccination of patients with diabetes are not clear. To the best of our knowledge, the side effects of the patients with diabetes were not well reported before. Evidence of these side effects is vitally important for health workers and diabetic patients which may help them to judge whether to receive the COVID-19 vaccination to reduce the risk of infection with SARS-CoV-2.

The Health Belief Model (HBM) is a model that interprets factors associated with health-related behaviors and attitudes, including perceived susceptibility, perceived severity, perceived benefit, perceived barriers, and action cues. It can be used to measure beliefs related to vaccination intentions and the prediction of vaccination behavior [23]. The model was once applied to interpret factors influencing influenza vaccination behavior in the Middle East [24], and to explain the predictors of maternal pertussis vaccination during pregnancy [25].

China has the most vaccinated doses of COVID-19 vaccines in the world, and the main vaccines used for SARS-CoV-2 are inactivated vaccines. As of 11 December 2021, 82.5% of China’s population are immunized against COVID-19 vaccines [26]. To fill the gap in the cognition of the COVID-19 vaccination uptake behavior and its side effects among diabetic patients, we intend to investigate the current situation of Chinese diabetes patients’ vaccination uptake behavior with the COVID-19 inactivated vaccine through a cross-sectional survey and to analyze the factors affecting their vaccination behavior with the HBM model theory.

## 2. Materials and Methods

### 2.1. Study Design

We conducted a cross-sectional survey of diabetic patients hospitalized in two affiliated hospitals in Changzhi City, Shanxi Province (Peace Hospital Affiliated with Changzhi Medical College and Heji Hospital) through face-to-face interviewing between June and October 2021.

Before the survey began, an expert panel composed of epidemiology professors, statisticians, and clinically experienced endocrinologists reviewed the questionnaire and determined its content effectiveness based on the fit of each statement in the questionnaire to the corresponding theoretical variables. Then, the questionnaire was revised according to the revision comments from the expert panel and the diabetic patients who attended the pilot study.

The questionnaire consists of three major items: (1) demographic information and health status; (2) perception of the risk of SARS-CoV-2 infection and cognition of COVID-19 vaccine; and (3) COVID-19 vaccination uptake behavior.

The demographic and background information and health status include gender, age, ethnicity, education level, marital status, occupation, diabetes prevalence, family history, etc. The questionnaire also includes the current physical condition of diabetic patients, including whether they are suffering from other chronic diseases (such as hypertension, hyperlipidemia, coronary heart disease, stroke, fatty liver, etc.), diabetic complications (diabetic nephropathy, diabetic retinopathy, etc.), diabetic foot, diabetic neuropathy, and blood sugar control effect (fasting blood sugar kept less than 13.9 mmol/L and having a stable blood sugar control level through lifestyle adjustment or drug treatment).

The primary outcome event of this study was the behavior of the subjects receiving the COVID-19 vaccine. The candidate options were as follows: 1 (vaccinated) and 0 (not vaccinated).

We used the HBM model to investigate the behavior of diabetes patients toward COVID-19 vaccination and analyze the factors affecting the uptake of COVID-19 vaccination behavior. The model mainly includes five aspects of information: First, the perceived susceptibility to SARS-CoV-2; Second, the perceived severity of COVID-19; Third, the perceived benefits of the COVID-19 vaccine; Fourth, the perceived barriers to COVID-19, and the fifth, the action clues of COVID-19 vaccination. The candidate options for the above question are as follows: 1 (strongly disagree), 2 (disagree), 3 (neutral), 4 (agree), and 5 (strongly agree).

### 2.2. Inclusion and Exclusion Criteria

Patients diagnosed with type 1 and 2 diabetes and hospitalized in the two above-mentioned affiliated hospitals during the study period, aged older than 18 years, who agreed to attend this study and provided written informed consent were included in this study. Participants with apparent mental illness or who have taken drugs for mental illness in the past three months, with apparent symptoms of dementia, or who cannot communicate in language were excluded.

### 2.3. Sample Size Calculation

Sample size was determined in advance according to the formula for estimating the total rate parameter in a cross-sectional study, α is the significance level, which is taken as 0.05, and Z1-α/2 is 1.96; *p* was taken as the estimated COVID-19 vaccination uptake rate in diabetic patients (39.0%) (based on peer study to COVID-19 vaccination uptake behavior of patients with chronic diseases [27]), and δ is the allowable error which was taken as 0.05. Therefore, the calculated minimum sample size of this project was 451 participants, considering a 10% of non-response rate and 90% of the questionnaire qualified rate.

### 2.4. Statistical Analysis

People with diabetes were divided into two groups based on their vaccination behaviors: those who had been vaccinated against COVID-19 and those who had not been vaccinated. Logistic regression models were used to calculate the crude odds ratios (c OR s) with COVID-19 vaccination behavior as dependent variables, sociodemographic characteristics, lifestyle and health status, and information related to HBM as independent variables. Adjusted odds ratios (a OR) and 95% CI were obtained after adjusting for *p* < 0.05 variables in univariate analysis to assess the association between our variable of interest and the dependent variable in the HBM model. All analyses were performed based on SPSS V25.0 software (IBM, Armonk, NY, USA).

## 3. Results

### 3.1. Baseline Characteristics

Of the 879 people who approached to participate in the study, 645 completed the survey with a response rate of 73.4%. Thirty-five people were excluded from the original sample because they rejected to participate in the study.

Among the 645 respondents, majority of the respondents were male (*n* = 361, 56.0%), aged 50–69 years (*n* = 350, 54.3%), Han nationality (*n* = 633, 98.1%), had a high school education or below (*n* = 379, 58.8%), married or living together (*n* = 569, 88.2%), farmer (n = 268, 41.6%) or other occupations (*n* = 234, 36.3%), per capita monthly income <2000 Yuan (US $ 320) (*n* = 263, 40.8%). Regarding the characteristics of lifestyle and health status of the participants, 61.6% (*n* = 397) and 64.7% (*n* = 417) of the respondents had smoking and drinking behavior; 74.9% (*n* = 483) of the respondents had chronic diseases other than diabetes, 56.6% (*n* = 417) had other chronic diseases. A total of 365 had diabetic comorbidities, 56.4% (*n* = 364) of respondents had a family history of diabetes, 31.0% (*n* = 200) of respondents had been diagnosed with diabetes for more than 10 years, and 6.5% of respondents reported the fasting blood glucose was >13.9 mmol/L, and nearly 50% of the respondents reported having a postprandial blood glucose >11.1 mmol/L in the most recent test (Table 1 and Table 2). 

### 3.2. COVID-19 Vaccination HBM Variables

Regarding the awareness of the COVID-19, 79.7% (514/645) of the respondents believe that they have a high risk of contracting SARS-CoV-2, and 39.4% (254/645) of the respondents believe that the COVID-19 syndrome is severe; Regarding the benefits of vaccination, 42.2% (272/645) of respondents believed that vaccination against COVID-19 could reduce the risk of SARS-CoV-2 infection, 38.0% (245/645) believed that vaccination could also reduce the risk of transmission to others, and 8.8% (57/645) of respondents, feel that vaccination is beneficial to themselves and others. In terms of perceived impairment, 61.2% (395/645) of respondents are concerned about the safety of COVID-19 vaccination, and 48.1% (310/645) were concerned about the side effects of vaccination. In addition, 26.8% (173/645) self-reported that the vaccination of their relatives had an impact on their own vaccination behavior, and 62.6% (404/483) of the respondents were agreed with the doctor’s statement (“vaccination can reduce the risk of SARS-CoV-2 infection”), and 50.2% (324/483) of the respondents reported that they would choose to receive the COVID-19 vaccination if they obtained the guidance and advice on the COVID-19 vaccination through the Internet/media platform (Table 3).

### 3.3. COVID-19 Vaccination Status and Related Factors

Of the diabetic patients who participated in this survey, 25.2% (162/645) (95% CI: 21.8–28.6%) were vaccinated against COVID-19.

Univariate logistic regression analysis showed that compared with unvaccinated participants, the participants who uptook the COVID-19 vaccine were most likely engaged in public utilities (vs. farmers) and had higher monthly income (≥5000 Yuan (vs. <2000 Yuan)), and those with relatively stable glycemic control (vs. unstable glycemic control).

Meanwhile, COVID-19 vaccinated people were significantly less likely to be female (vs. males), live in rural areas (vs. urban), smoke and drink alcohol, have other chronic diseases and diabetic complications than non-vaccinated people, and have a family history of diabetes, report the most recently measured fasting blood glucose greater than 13.9 mmol/L (vs. <7 mmol/L) (Table 1 and Table 2).

Regarding the variables in the HBM of COVID-19 vaccination, believing that they were at higher risk of contracting SARS-CoV-2 (perceived sensitivity), believing that the COVID-19 syndrome was severe (perceived severity), believing that vaccination would reduce SARS-CoV-2 risk of infection, the perception that vaccination is beneficial to self and others (perceived benefits of vaccination), the impact of vaccination by relatives on their vaccination behavior, and claims to doctors that “vaccination reduces the risk of SARS-CoV-2 infection” Identity (action cues) were positively correlated with the COVID-19 vaccination behavior of diabetes respondents.

Concerns about the safety of the COVID-19 vaccine and the side effects of vaccination (perceived impairment) were inversely associated with the COVID-19 vaccination behavior of diabetes respondents (Table 3).

The perception of the severity of the COVID-19 syndrome (a OR 3.67, 95%CI 1.88–7.17; *p* < 0.001), the conception of substantial reduction of the SARS-CoV-2 infection risk after vaccination (a OR 3.48, 95%CI 1.80–6.73; *p* < 0.001), feeling of the beneficial effects of vaccination on self and others (a OR 4.53, 95%CI 1.71–11.99; *p* = 0.002), fear of the possible adverse impact of COVID-19 vaccination on health conditions (a OR 0.18, 95%CI 0.09–0.35; *p* < 0.001), the positive effect of self-reporting relatives’ vaccination behavior toward self’s vaccination (a OR 5.68, 95%CI 2.83–11.39; *p* < 0.001) were independently associated with SARS-CoV-2 vaccination behavior of diabetes respondents (Table 4).

### 3.4. Adverse Reactions after COVID-19 Vaccination

Of the 162 vaccine receivers, 13.6% (22/162) were self-reported to have adverse reactions to vaccination within one month after vaccination. All those were minor reactions, and the most common adverse reaction was pain at the injection site (36.7%), followed by fatigue, headache, dizziness or drowsiness (23.3%), muscle pain or joint pain (20.0%), and other minor reactions at the injection site such as red, swollen, pruritus, callous or rash (10.0%), pruritus at non-injection site (6.7%), and nausea, vomitive or diarrhea (3.3%), no serious adverse vaccination reactions were reported.

## 4. Discussion

To the best of our knowledge, this survey is the first to identify lower COVID-19 vaccination rates among diabetic populations in China, representing the latest estimates of COVID-19 vaccination rates among diabetic populations in central China. Moreover, this study is also the first to use HBM model analysis to reveal the main factor that may hinder the behavior of COVID-19 vaccination in patients with diabetes. The findings of this study will enhance medical staff’s understanding of the COVID-19 vaccination behavior and related factors in diabetic patients, and showed that the COVID-19 vaccination proportion was low in diabetic patients in rural areas and female participants. Hence health education and behavioral intervention should be carried out for this population to enhance their awareness of COVID-19 vaccination.

Vaccination against COVID-19 is the most economical and effective way to prevent and control the pandemic. It is also one of the most comprehensive measures to prevent SARS-CoV-2 infection and severe COVID-19 cases. In this study, the vaccination rate of the COVID-19 vaccine among Chinese diabetic patients was 162/645 (25.2%), which was higher than that in an Indian study (21.5%) [28] while lower than that in Saudi Arabia (34.7%) [16]. The COVID-19 vaccination rate for diabetic patients in China is also significantly lower than that in cancer patients (35.5%) [29], in multiple sclerosis patients (53.5%) [30], and in HIV population in China (43.7%) and in HIV population in the United States (64.0%) [31,32]. However, there is a gap between willingness to vaccinate and actual uptake behavior of vaccination. At present, the vaccination against COVID-19 is still in progress and the vaccination situation keeps changing, thus the willingness and behavior to be vaccinated of the diabetic population might change in the future.

Multivariable logistic regression was used to identify the factors associated with COVID-19 vaccination behavior in diabetic patients. First, for sociological background information, this study found that males, people in public institutions, people living in cities and towns, and those with higher economic income (monthly income > 5000 YUAN) were more likely to vaccinate against COVID-19. This may be due to males’ higher perception of risk than females, thus more willing to vaccinate against the COVID-19. This is consistent with the results of Ania Wisniak’s [33] study on COVID-19 vaccination rates among adults in Geneva. The vaccination rate of the COVID-19 vaccine is also higher among those working in public institutions or institutions. The possible reason might be the characteristics of their work environment (such as those in public transportation, customs, doctors, teachers, etc.). There are more administrative constraints from the management department as they are normally exposed to a higher risk of contracting SARS-CoV-2, resulting in the relatively higher vaccination rate for the COVID-19 vaccine. The high vaccination rate of COVID-19 in urban residents may be due to the higher concentration of the urban population and the higher level of community health services for vaccination, promoting the vaccination rate of diabetic patients. People with higher economic income are more receptive to new things and external information and are more likely to receive the vaccination guidance issued by the management department to receive the COVID-19 vaccine in time. Our results suggested that China must increase education and publicity on the prevention and control of the COVID-19 epidemic for rural residents in the future and convey more professional health guidance and advice to increase their vaccination rate. At the same time, it is emphasized that the SARS-CoV-2 is also transmitted through air contact, and the mutant strains continue emerging. Even if they are not employees of public institutions, they should respond to the state’s call and actively vaccinate against the COVID-19. Second, the characteristics related to lifestyle and health status, such as smoking, drinking, family history of diabetes and other chronic diseases (such as hyperlipidemia, hypertension, coronary heart disease, fatty liver, stroke, etc.) and fasting blood sugar > 13.9 mmol/L were negatively associated with vaccination behavior. It also demonstrated that the people mentioned above may hesitate to receive the COVID-19 vaccine because of their lifestyle or health status, thus not actively receiving the COVID-19 vaccine. However, the above characteristics are not contraindications to the COVID-19 vaccination except for fasting blood glucose > 13.9 mmol/L. Therefore, it is necessary to strengthen in-depth publicity and education in the future to make people with the above characteristics and complications aware of whether they are suitable for the COVID-19 vaccine to reduce the risk of contracting SARS-CoV-2 by vaccinating against COVID-19.

Based on the HBM model, we also investigated the factors that affect the behavior of the respondents to receive the COVID-19 vaccine. Firstly, in this study, we claimed that the vaccination behavior of diabetic patients was positively correlated with a high risk of contracting SARS-CoV-2 and severe COVID-19 syndrome. While few of these people who agreed with this statement had been vaccinated against COVID-19. Similar to vaccination situation, diabetic patients’ perception of epidemic severity is a direct motivation to increase vaccination intention [34]; Secondly, the vaccination behavior of diabetic patients is positively correlated with the assertion that vaccination is beneficial for themselves and others. Previous studies have shown that the awareness of the benefits of vaccination among diabetic patients is a contributing factor to increase vaccination rates [34]; Thirdly, we found that individuals who were concerned about the safety of COVID-19 vaccination and adverse effects after vaccination had significantly higher rates of vaccination against COVID-19. This may be related to the lack of knowledge about the safety and side effects of vaccination in diabetic patients, which is in line with a published study of vaccination in diabetic patients [35]. In the future, the health department can provide reports on the safety and side effects of COVID-19 vaccination in patients with diabetes in order to alleviate the concerns of diabetic patients; Fourthly, most people do not agree that relatives’ vaccination behavior will affect them, but they do agree with what clinical doctor’s explanation about vaccination recommendations. This is similar to the results of previous vaccination studies, in which the research subjects regard medical staff as the authority and they had relatively high compliance with the advice raised by the medical practitioners, regarding the recommendation on vaccination for COVID-19 [36]. It also suggested the significant role of medical workers in promoting the vaccination in order to increase the willingness of people with diabetes to get vaccinated.

The evidence of adverse reactions to vaccination is a crucial factor for the respondents to receive vaccination. We showed that concern about the vaccination-related adverse effects was an independent factor of COVID-19 vaccination uptake in people with diabetes. This concern is understandable given the novelty of COVID-19 vaccines, but current reports of adverse effects following the COVID-19 vaccine are mild [37,38]. Our survey found that of 162 vaccine recipients, 13.6% (22/162) were self-reported to have adverse reactions to vaccination within one month after vaccination. All those were minor reactions such as pain at the injection site, fatigue, etc. No serious adverse vaccination reactions were reported. It is lower than the proportions in other studies, such as the proportion of adverse reactions reported in the HIV population (13.6% vs. 29.4%) [39] and healthy adults who were vaccinated against COVID-19 (13.6% vs. 15.0%) [40]. This also shows that patients with diabetes can be vaccinated without serious adverse reactions after being vaccinated against COVID-19. However, it is still necessary to strengthen the long-term monitoring of patients with diabetes after vaccination against COVID-19 in order to better clarify the mid- and long-term effects of vaccination in patients with diabetes.

One of the strengths of this study is that this study is the first survey conducted on the vaccination behavior of diabetic patients, which may promote the vaccination behavior of patients with diabetes in other regions of China and the rest of the world. Besides, this study is also the first to use the HBM model analysis to reveal major factors that may hinder the behavior of COVID-19 vaccination in diabetic patients. These findings can help the public health sectors to understand the COVID-19 vaccination behavior and related factors in diabetes patients, providing a basis for targeted educational and behavioral interventions. Furthermore, medical researchers should accelerate the conduction of more safety studies on COVID-19 vaccination and actively monitor its side effects as well as medium-and long-term effects to better guide potential theory-based behavioral interventions.

Our study has the following limitations: Firstly, as this study was conducted among hospitalized diabetic populations, it was not representative of all diabetes patients. Thus, we will recruit participants from non-hospitalized diabetes patients in the same period as a control group to compare results in a future study. Secondly, we compressed the five-point scale (strongly disagree, disagree, neutral, agree, and strongly agree) into a binary answer of “yes/no”, which may have led to some underlying information being missing, and a more complex analysis should be used in the future to explain all five options on the ordinal scale. Thirdly, this study only reported the short-term occurrence of the adverse reactions to COVID-19 vaccination with a relatively small sample size in diabetic patients. Future studies may focus on the long-term adverse reaction effect of COVID-19 vaccination with a larger sample of diabetic patients. Lastly, our study used a cross-sectional survey research design. Thus causal relationships between predictor and outcome variables could not be determined. 

## 5. Conclusions

The safety of the COVID-19 vaccine and the side effects of vaccination are the main factors influencing the vaccination behavior of diabetic patients based on the theory of HBM. Healthcare workers should provide more publicity and educational information on vaccine administration safety and protective effects to improve vaccination behaviors among people with diabetes. Also, the general public should be educated sufficiently about the safety of vaccination for vaccination campaigns to be carried out effectively among the diabetic population.

## Figures and Tables

**Table 1:** The demographics characteristics of diabetic participants. (*n* = 645).

Characteristic	COVID-19 Vaccination			Gotten vs. Did Not Get c OR ^a^ (95% CI)	*p*-Value
	Overall (*n* = 645)	Gotten (*n* = 162)	Did Not Get (*n* = 483)		
Gender					
Male	361 (56.0)	109 (67.3)	252 (52.2)	1.0	Ref ^b^
Female	284 (44.0)	53 (32.7)	231 (47.8)	0.53 (0.37–0.77)	0.001
Age group (years)					
18–39	68 (10.5)	18 (11.1)	50 (10.4)	1.0	Ref
40–49	115 (17.8)	37 (22.8)	78 (16.1)	1.32 (0.68–2.56)	0.42
50–59	187 (29.0)	44 (27.2)	143 (29.6)	0.86 (0.45–1.61)	0.63
60–69	163 (25.3)	31 (19.1)	132 (27.3)	0.65 (0.34–1.27)	0.21
≥70	112 (17.4)	32 (19.8)	80 (16.6)	1.11 (0.57–2.19)	0.76
Nationality					
Han	633 (98.1)	158 (97.5)	475 (98.3)	1.0	Ref
Non-Han	12 (1.9)	4 (2.5)	8 (1.7)	1.50 (0.45–5.06)	0.51
Education level					
Below high school	379 (58.8)	91 (56.2)	288 (59.6)	1.0	Ref
High school	122 (18.9)	27 (16.7)	95 (19.7)	0.90 (0.55–1.47)	0.67
College and above	144 (22.3)	44 (27.2)	100 (20.7)	1.39 (0.91–2.13)	0.13
Marital status					
Unmarried, Divorced, Separated, or Widowed	76 (11.8)	17 (10.5)	59 (12.2)	1.0	Ref
Married or Cohabitation	569 (88.2)	145 (89.5)	424 (87.8)	1.19 (0.67–2.10)	0.56
Occupation type					
Farmer	268 (41.6)	58 (35.8)	210 (43.5)	1.0	Ref
Public institution Personnel	143 (22.2)	53 (32.7)	90 (18.6)	2.13 (1.36–3.33)	0.001
Others	234 (36.3)	51 (31.5)	183 (37.9)	1.01 (0.66–1.54)	0.97
Residence					
Urban	377 (58.4)	110 (67.9)	267 (55.3)	1.0	Ref
Rural	268 (41.6)	52 (32.1)	216 (44.7)	0.58 (0.40–0.85)	0.005
Monthly personal income (Chinese Yuan ^c^)					
<2000	263 (40.8)	58 (35.8)	205 (42.4)	1.0	Ref
2000–3499	178 (27.6)	36 (22.2)	142 (29.4)	0.90 (0.56–1.43)	0.65
3500–4999	131 (20.3)	38 (23.5)	93 (19.3)	1.44 (0.90–2.33)	0.13
≥5000	73 (11.3)	30 (18.5)	43 (8.9)	2.47 (1.42–4.27)	0.001

Data are shown as n (%). ^a^ c OR: crude odds ratio; ^b^ Ref: reference; ^c^ A currency exchange rate of 1 Chinese Yuan = US $0.16 is applicable.

**Table 2:** Lifestyle and health conditions of diabetic participants. (*n* = 645).

Condition	COVID-19 Vaccination			Gotten vs. Did Not Get c OR ^a^ (95% CI)	*p*-Value
	Overall (*n* = 645)	Gotten (*n* = 162)	Did Not Get (*n* = 483)		
Current smoker					
No	248 (38.4)	103 (63.6)	145 (30.0)	1.0	Ref ^b^
Yes	397 (61.6)	59 (36.4)	338 (70.0)	0.25 (0.17–0.36)	<0.001
Current drinker					
No	228 (35.3)	107 (66.0)	121 (25.1)	1.0	Ref
Yes	417 (64.7)	55 (34.0)	362 (74.9)	0.17 (0.12–0.25)	<0.001
Other Chronic diseases ^#^					
No	162 (25.1)	95 (58.6)	67 (13.9)	1.0	Ref
Yes	483 (74.9)	67 (41.4)	416 (86.1)	0.11 (0.08–0.17)	<0.001
Complications of diabetes *					
No	280 (43.4)	121 (74.7)	159 (32.9)	1.0	Ref
Yes	365 (56.6)	41 (25.3)	324 (67.1)	0.17 (0.11–0.25)	<0.001
Blood glucose level controlled ^※^					
No	312 (48.4)	50 (30.9)	262 (54.2)	1.0	Ref
Yes	333 (51.6)	112 (69.1)	221 (45.8)	2.66 (1.82–3.88)	<0.001
Family history of diabetes					
No	281 (43.6)	108 (66.7)	173 (35.8)	1.0	Ref
Yes	364 (56.4)	54 (33.3)	310 (64.2)	0.28 (0.19–0.41)	<0.001
Time since diabetes diagnosis (years)					
≤1	180 (27.9)	39 (24.1)	141 (29.2)	1.0	Ref
2–10	265 (41.1)	69 (42.6)	196 (40.6)	1.27 (0.81–1.99)	0.29
>10	200 (31.0)	54 (33.3)	146 (30.2)	1.34 (0.83–2.15)	0.23
Fasting blood glucose in the most recent episode of testing (mmol/L)					
<7	236 (36.6)	67 (41.4)	169 (35.0)	1.0	Ref
7–13.9	367 (56.9)	91 (56.2)	276 (57.1)	0.83 (0.58–1.20)	0.33
>13.9	42 (6.5)	4 (2.5)	38 (7.9)	0.27 (0.09–0.77)	0.02
Postprandial blood glucose in the most recent episode of testing (mmol/L)					
<10	221 (34.3)	62 (38.3)	159 (32.9)	1.0	Ref
10–11.1	117 (18.1)	30 (18.5)	87 (18.0)	0.88 (0.53–1.47)	0.64
>11.1	307 (47.6)	70 (43.2)	237 (49.1)	0.76 (0.51–1.13)	0.17

Data are shown as n (%). ^a^ c OR: crude odds ratio; ^b^ Ref: reference; **^#^**: refer to Hypertension; Hyperlipidemia; Coronary Heart Disease; Cerebral Stroke; Fatty liver. *: refer to Diabetic nephropathy; Diabetic retinopathy; Diabetic foot; Diabetic neuropathy. ^※^: Through lifestyle adjustment or drug treatment, fasting blood glucose ≤ 13.9 mmol/L.

**Table 3:** Variables about HBM among diabetic participants. (*n* = 645).

Variable	COVID-19 Vaccination			Gotten vs. Did Not Get c OR ^a^ (95% CI)	*p*-Value
	Overall (*n* = 645)	Gotten (*n* = 162)	Did Not Get (*n* = 483)		
Have you gotten the COVID-19 vaccination					
No	483 (74.8)	0 (0)	483 (100)	N/A ^b^	N/A
Yes	162 (25.2)	162 (100)	0 (0)	N/A	N/A
Perceived susceptibility					
The risk of acquiring SARS-CoV-2 is high					
No (strongly disagree or disagree or neutral)	131 (20.3)	19 (11.7)	112 (23.2)	1.0	Ref ^c^
Yes (agree or very agree)	514 (79.7)	143 (88.3)	371 (76.8)	2.27 (1.35–3.83)	0.002
Perceived severity					
The COVID-19 syndrome is severe					
No (strongly disagree or disagree or neutral)	391 (60.6)	62 (38.3)	329 (68.1)	1.0	Ref
Yes (agree or very agree)	254 (39.4)	100 (61.7)	154 (31.9)	3.45 (2.38–4.99)	<0.001
Perceived benefits					
Vaccination reduces the risk of infection					
No (strongly disagree or disagree or neutral)	373 (57.8)	54 (33.3)	319 (66.0)	1.0	Ref
Yes (agree or very agree)	272 (42.2)	108 (66.7)	164 (34.0)	3.89 (2.67–5.67)	<0.001
Vaccination reduces the risk of transmission to other people					
No (strongly disagree or disagree or neutral)	400 (62.0)	103 (63.6)	297 (61.5)	1.0	Ref
Yes (agree or very agree)	245 (38.0)	59 (36.4)	186 (38.5)	0.92 (0.63–1.32)	0.64
Vaccination is good for yourself and others					
No (strongly disagree or disagree or neutral)	588 (91.2)	125 (77.2)	463 (95.9)	1.0	Ref
Yes (agree or very agree)	57 (8.8)	37 (22.8)	20 (4.1)	6.85 (3.84–12.22)	<0.001
Perceived barriers					
As a person with diabetes, I worried about the safety of the COVID-19 vaccination					
No (strongly disagree or disagree or neutral)	250 (38.8)	104 (64.2)	146 (30.2)	1.0	Ref
Yes (agree or very agree)	395 (61.2)	58 (35.8)	337 (69.8)	0.24 (0.17–0.35)	<0.001
Worried about side effects of vaccination					
No (strongly disagree or disagree or neutral)	335 (51.9)	105 (64.8)	230 (47.6)	1.0	Ref
Yes (agree or very agree)	310 (48.1)	57 (35.2)	253 (52.4)	0.49 (0.34–0.71)	<0.001
Action clues					
Relatives’ vaccination action will affect your vaccination uptake behavior					
No (strongly disagree or disagree or neutral)	472 (73.2)	74 (45.7)	398 (82.4)	1.0	Ref
Yes (agree or very agree)	173 (26.8)	88 (54.3)	85 (17.6)	5.57 (3.78–8.21)	<0.001
Believe in the doctor’s statement that vaccination can reduce the risk of infection					
No (strongly disagree or disagree or neutral)	241 (37.4)	41 (25.3)	200 (41.4)	1.0	Ref
Yes (agree or very agree)	404 (62.6)	121 (74.7)	283 (58.6)	2.09 (1.40–3.11)	<0.001
Advice on vaccination from the internet/media					
No (strongly disagree or disagree or neutral)	321 (49.8)	71 (43.8)	250 (51.8)	1.0	Ref
Yes (agree or very agree)	324 (50.2)	91 (56.2)	233 (48.2)	1.38 (0.96–1.97)	0.08

Data are shown as n (%). ^a^ c OR: crude odds ratio; ^b^ N/A: not applicable; c OR was not calculated for this item; ^c^ Ref: reference.

**Table 4:** Factors associated with COVID-19 vaccination uptake behavior among diabetic participants (*n* = 645).

Variable	a OR ^a^ (95%CI)	*p*-Value
Perceived susceptibility		
The risk of acquiring SARS-CoV-2 is high		
No (strongly disagree or disagree or neutral)	1.0	Ref ^b^
Yes (agree or very agree)	0.98 (0.44–2.16)	0.96
Perceived severity		
The COVID-19 syndrome is severe		
No (strongly disagree or disagree or neutral)	1.0	Ref
Yes (agree or very agree)	3.67 (1.88–7.17)	<0.001
Perceived benefits		
Vaccination reduces the risk of infection		
No (strongly disagree or disagree or neutral)	1.0	Ref
Yes (agree or very agree)	3.48 (1.80–6.73)	<0.001
Vaccination reduces the risk of transmission to other people		
No (strongly disagree or disagree or neutral)	1.0	Ref
Yes (agree or very agree)	1.23 (0.62–2.44)	0.55
Vaccination is good for yourself and others		
No (strongly disagree or disagree or neutral)	1.0	Ref
Yes (agree or very agree)	4.53 (1.71–11.99)	0.002
Perceived barriers		
As a person with diabetes, I worried about the safety of the vaccination		
No (strongly disagree or disagree or neutral)	1.0	Ref
Yes (agree or very agree)	0.18 (0.09–0.35)	<0.001
Worried about side effects of vaccination		
No (strongly disagree or disagree or neutral)	1.0	Ref
Yes (agree or very agree)	0.59 (0.28–1.24)	0.16
Action clues		
Relatives’ vaccination action will affect your vaccination uptake behavior		
No (strongly disagree or disagree or neutral)	1.0	Ref
Yes (agree or very agree)	5.68 (2.83–11.39)	<0.001
Believe in the doctor’s statement that vaccination can reduce the risk of infection		
No (strongly disagree or disagree or neutral)	1.0	Ref
Yes (agree or very agree)	1.57 (0.73–3.38)	0.25
Advice on vaccination from the internet/media		
No (strongly disagree or disagree or neutral)	1.0	Ref
Yes (agree or very agree)	0.50 (0.25–1.02)	0.06

^a^ a OR: adjusted odds ratio; odds ratios were obtained by fitting a single logistic regression model involving an independent variable of interest and all background variables listed in Table 1 and Table 2 with *p* < 0.05 in univariate analysis; ^b^ Ref: reference.

## Data Availability

Not applicable.
